# Evidence for uteroplacental malperfusion in fetuses with major congenital heart defects

**DOI:** 10.1371/journal.pone.0226741

**Published:** 2020-02-05

**Authors:** Julia Binder, Silvia Carta, Julene S. Carvalho, Erkan Kalafat, Asma Khalil, Basky Thilaganathan

**Affiliations:** 1 Fetal Medicine Unit, St George's University Hospitals NHS Foundation Trust and Molecular & Clinical Sciences Research Institute, St George's University of London, London, England, United Kingdom; 2 Department of Obstetrics and Fetomaternal Medicine, Medical University of Vienna, Vienna, Austria; 3 Brompton Centre for Fetal Cardiology, Royal Brompton Hospital, London, England, United Kingdom; 4 Ankara University Faculty of Medicine, Department of Obstetrics and Gynecology, Ankara, Turkey; 5 Middle East Technical University, Department of Statistics, Ankara, Turkey; University of Mississippi Medical Center, UNITED STATES

## Abstract

**Aims:**

Fetuses affected by congenital heart defects (CHD) are considered to be at increased risk of fetal growth restriction and intrauterine demise. Whether these risks are a direct consequence of fetal CHD or a result of associated uteroplacental dysfunction is not evident from the data of recent studies. The aim of this study was to investigate the prevalence of uteroplacental dysfunction reflected by abnormal uterine artery Doppler indices and reduced fetal growth in CHD pregnancies.

**Methods:**

This is a retrospective case-control study including singleton pregnancies referred for detailed fetal cardiac assessment subsequently diagnosed with or without CHD. Mid-trimester uterine artery Doppler assessment at 20–24 weeks as well as third trimester fetal biometry and arterial Doppler pulsatility indices (PI) were performed. All fetal biometry were converted into centiles and Doppler values to multiples of median (MoM) to adjust for physiological changes with gestation.

**Results:**

The study included 811 pregnancies including 153 cases where the fetus was diagnosed with CHD. Mid-pregnancy uterine artery PI was significantly higher in women with fetal CHD compared to controls (0.90MoM vs 0.83MoM; p = 0.006). In the third trimester, median centiles for fetal head circumference (45.4 vs 57.07; p<0.001), abdominal circumference (51.17 vs 55.71; p = 0.014), estimated fetal weight (33.6 vs 56.7; p<0.001) and cerebroplacental ratio (CPR: 0.84MoM vs 0.95MoM; p<0.001) were significantly lower in fetuses with CHD compared to controls. The percentage of small for gestational age births <10^th^ centile (24.0% vs 10.7%; <0.001) and low CPR <0.6MoM (11.7% vs 2.5%; p<0.001) were significantly higher in the fetal CHD cohort.

**Conclusions:**

Mid-pregnancy uterine artery resistance is increased and subsequent fetal biometry reduced in pregnancies with CHD fetuses. These findings suggest that fetal CHD are associated with uteroplacental dysfunction, secondary to impaired maternal uteroplacental perfusion resulting in relative fetal hypoxaemia and reduced fetal growth.

## Introduction

Congenital heart defects (CHD) affect around 1% of live births and are considered the leading cause of neonatal mortality due to birth defects [1]. Although the survival rates for fetal CHD have improved, the risk of prenatal and postnatal brain abnormalities and neurodevelopmental delay in fetuses and neonates with CHD continues to be a significant problem [2, 3]. A number of studies have provided data suggesting that the risk of neurological impairment is not only a result of hypoxia during cardiac surgery [4], but also highlighted the link between reduced fetal middle cerebral artery (MCA) pulsatility index (PI), reduced fetal cerebral oxygenation and a reduction in the brain volume [2, 5–7]. Consistent with fetal hypoxemia in CHD, other studies have suggested the presence of placental dysfunction with significantly higher umbilical artery (UA) Doppler PI and reduced fetal size [2, 5, 6].

Recent data suggest uteroplacental malperfusion as an additional or even underlying mechanism in fetuses with CHD describing decreased levels of placental like growth factor (PLGF) in isolated major CHD in the first trimester, indicating impaired placentation [8, 9]. Moreover soluble fms-like tyrosine kinase 1 (sFlt-1), vascular endothelial growth factor A and soluble endoglin, markers of antiangiogenesis, were expressed in higher amounts in fetal heart tissue and fetal cord blood when a CHD was present [10]. Furthermore maternal serum of women carrying a fetus with a CHD showed decreased PLGF and increased sFlt1 levels, which is hypothesized to be due to impaired placental angiogenesis [10].

Additionally, a subsequent prospective study evaluating uteroplacental perfusion in fetuses with CHD in the first trimester was also able to demonstrate placental dysfunction represented by low pregnancy-associated plasma protein A (PAPP-A) and placental like growth factor (PlGF). However, this study did not assess uteroplacental perfusion reflected by uterine artery doppler indices [11]. The aim of this study was to ascertain whether pregnancies complicated by fetal CHD exhibited Doppler features consistent with maternal uteroplacental malperfusion and ultrasound/Doppler evidence of fetal growth restriction.

## Materials and methods

### Study population and patient characteristics

The Wandsworth Research Ethics Committee waived the requirement for review and approval by a Research Ethics Committee and for patient consent to access of identified clinical data by the lead author.

This is a retrospective cross sectional case control study, including data from all pregnant women referred to the Fetal Medicine Unit at St George’s Hospital (SGH) for detailed fetal cardiology assessment after 20 weeks’ gestation, between January 2009 and January 2016. Major congenital fetal heart defect was defined as a structural heart defect that was likely to require surgical repair. The cases also had further third trimester fetal assessment, which included fetal biometry, UA and MCA Doppler evaluation by a fetal medicine specialist. Uterine Artery (UtA) Doppler assessment was done in the second trimester after referral to SGH as per unit protocol to screen for preeclampsia and intrauterine growth restriction.

Control pregnancies underwent the same standard of cardiac and fetal medicine assessment due to family history of fetal heart abnormalities, suspected fetal heart abnormality or medication intake that might affect normal formation of the fetal heart, but had a final prenatal and postnatal diagnosis of normal heart structure and function. The exclusion criteria consisted of chromosomal or genetic abnormalities, minor cardiac defects, isolated venous system anomalies, prenatal fetal cardiac intervention/therapy, non-structural cardiac abnormalities (such as arrhythmias and cardiomegaly), extra-cardiac abnormalities, fetal infections causing hyperdynamic circulation, multiple pregnancies and maternal conditions that might affect fetal hemodynamics, growth or placental vascularization (such as hypertensive disorders, preeclampsia, thyroid disorders, autoimmune disorders and diabetes). Prenatal invasive testing and chromosomal testing were offered to all pregnant women diagnosed with a major congenital heart defect. Postnatal confirmation of the cardiac defect detected during pregnancy was performed by a pediatric/fetal cardiologist. Genetic testing was done postnatally if applicable, leading to exclusion of the study if chromosomal or genetic abnormalities were present.

CHD defects were divided into subgroups according to their expected pattern of blood supply to the brain as described by Masoller et al.[7] They were grouped into CHD with low placental blood delivered to the brain due to severe left ventricular outflow obstruction and CHD with mixed systemic and placental blood due to intracardial shunts[7].

## Ultrasound and Doppler technique

Fetal Doppler parameters were taken automatically from consecutive waveforms and the angle of insonation used was below 30°. The UA Doppler waveform was measured after sampling a free-floating portion of the umbilical cord using colour Doppler imaging. Consequently, the UA pulsatility index was calculated according to a standard protocol [12]. The MCA was identified at the level of its origin from the circle of Willis using colour Doppler after obtaining a transverse section of the fetal head. The MCA was sampled at the position where the vessel passes the sphenoid wing using Pulsed Doppler. The MCA PI was determined according to standard protocol [13]. The CPR was calculated as the ratio MCA-PI/UA-PI [14]. Uterine artery Doppler was assessed using colour Doppler to visualize the left and right uterine arteries at the level of the cross over with the external iliac artery. Pulsed-wave Doppler was applied to assess impedance to flow and the PI was measured over three consecutive waveforms. The mean value of the right and left UtA-PI was subsequently calculated [15]. All Doppler indices were converted into multiples of the median (MoM), correcting for gestational age in weeks [13–15]. EFW was calculated from the biparietal diameter, head and abdominal circumference and femur length using the Hadlock formula [16]. Birthweight (BW) and EFW values were converted into centiles [17]. Data on maternal characteristics, included age, body mass index and ethnic origin, obstetric and maternal medical history, ultrasound scans (biometry and Doppler evaluation in the second and third trimester), genetic testing, pregnancy outcomes and neonatal assessment, were retrieved for all included pregnant women using the Viewpoint obstetric ultrasound database (Viewpoint Inc., USA), Euroking K2 Maternity System and the neonatal records.

## Statistical analysis

Continuous variables were presented as median with interquartile ranges whereas binary variables were presented as numbers with percentages. Distribution assumptions for the variables were tested with Shapiro-Wilk test. Group comparisons were made with t-test when normal distribution assumptions were satisfied, otherwise Mann-Whitney-U test was used. The Fisher’s exact test was used for comparison of binary outcomes. P values below 0.05 were considered statistically significant. All group comparisons were made with two-tailed tests. The analysis was performed using the R for statistical computing software (Version 3.2.3, R Foundation for Statistical Computing, Vienna, Austria). The Kruskal Wallis Test was used to evaluate differences in UtA PI MoM in different subgroups of CHD.

## Results

The study included 811 pregnancies (153 CHD cases and 658 controls) with a total of 1622 scans (811 echocardiograms and 811 anomaly/growth scans). The spectrum of major fetal cardiac abnormalities in the study population is shown in Table 1.

**Table 1:** Types of congenital heart defects included in this study.

Type of congenital heart defect	Number
Ventricular septal defect	23
Transposition of great arteries (TGA)	13
Tetralogy of Fallot	21
Hypoplastic left heart syndrome	9
Aortic stenosis	2
Coarctation of the aorta	18
Congenitally Corrected TGA	1
Atrioventricular septal defect	10
Double outlet right ventricle	6
Double aortic arch	1
Ebstein´s anomaly	1
Pulmonary atresia	10
Pulmonary stenosis	8
Tricuspid Atresia	7
Interrupted aortic arch	1
Complex abnormalities*	22
**Total**	**153**

*Complex CHD not classifiable in any of the other above reported categories.

Maternal characteristics and pregnancy outcomes are shown in Table 2. In contrast to the controls that were all live born, in the CHD group there were 14 terminations of pregnancy, 6 stillbirths and 5 neonatal deaths. The diagnosis of CHD was confirmed after birth in the remaining 125 cases. The median gestational age (GA) at delivery was significantly lower in the CHD cases compared to controls (38.9 versus 39.9; p<0.001), as was the median birth weight centile (31.3 vs 44.1; p = 0.002) and the prevalence of small for gestational age births <10th centile (24.0% vs 10.7%; <0.001).

**Table 2:** Description of the study groups.

	Congenital heart defects (n = 153)	Controls (n = 658)	p value
**Maternal age (years)**	31.00 (28.00–34.00)	32.00 (28.00–36.00)	0.058
**Self-reported ethnicity**			0.042
Asian	18 (11.8)	108 (16.4)	
Black	17 (11.1)	79 (12.0)	
Caucasian	103 (67.3)	437 (66.4)	
Mixed	3 (2.0)	24 (3.6)	
Other	4 (2.6)	2 (0.3)	
Missing	8 (5.2)	8 (1.2)	
**Outcome of pregnancy**			<0.001
Intrauterine death	6 (3.9)	0 (0.0)	
Termination	14 (9.2)	0 (0.0)	
Live birth	125 (81.7)	658 (100.0)	
Neonatal death	5 (3.3)	0 (0.0)	
Missing	3 (1.9)	0 (0.0)	
**Gestational age at delivery (weeks)**	38.86 (36.96–39.57)	39.86 (39.00–40.86)	<0.001
**Mode of delivery**			0.013
Vaginal delivery	61 (39.9)	378 (57.4)	
Instrumental delivery	12 (7.8)	103 (15.7)	
Elective caesarean	29 (19.0)	93 (14.4)	
Emergency caesarean	19 (12.4)	74 (11.2)	
Missing	32 (20.9)	10 (1.5)	
**Neonatal sex (male)**	51.9 (%)	52.8 (%)	0.857
**Birth weight (grams)**	3120 (2642–3400)	3355 (3037–3690)	<0.001
**Birth weight centile**	31.31 (10.36–64.20)	44.13 (20.09–70.51)	0.002
**Birth weight <10**^**th**^ **centile**	24.0 (%)	10.7 (%)	<0.001

The values are presented as number (%) or Median (interquartile range). Group comparisons were made with Mann-Whitney-U, Chi-Square or Fisher’s exact where appropriate

**Fig 1:**
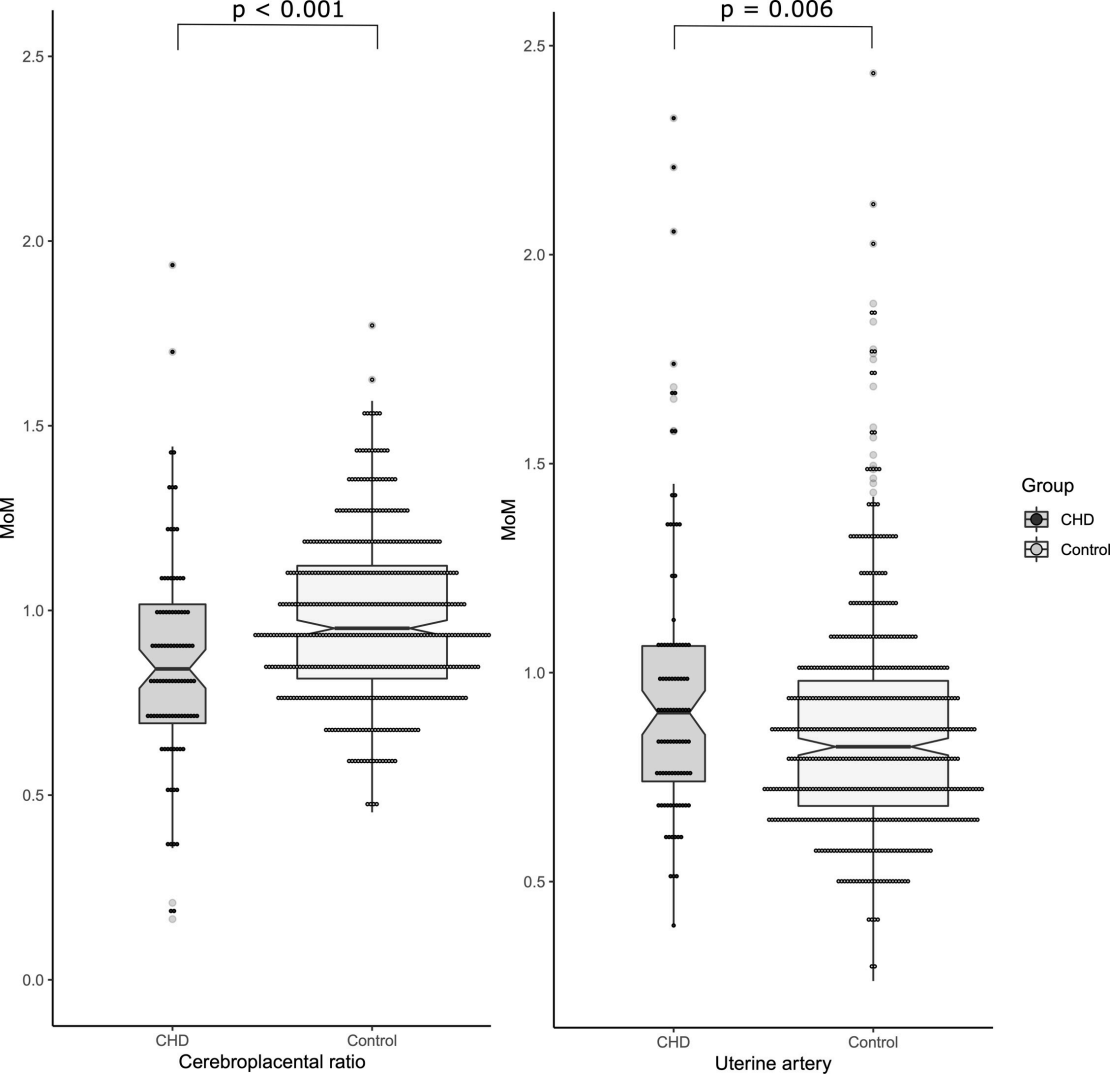
Scatter plots of the cerebroplacental ratio (Fig 1A) multiple of median (MoM) and the uterine artery MoM (Fig 1B) in the fetuses with congenital heart defects compared to normal fetuses. The dots represent individual values for patients. The middle bar represents the median for that sample and upper and lower bars represent interquartile ranges.

The details of the ultrasound and Doppler assessments are shown in Table 3. The median fetal head circumference (45.43 vs 57.07; p<0.001), abdominal circumference (51.17 vs 55.71; p = 0.014) and estimated fetal weight (EFW) centiles (33.60 vs 56.73; p<0.001) were significantly lower in fetuses with CHD compared to controls (Table 3). The mid-pregnancy median UtA PI MoM (0.90 vs 0.83; p = 0.006) and UA PI MoM (1.05 vs 0.97; p<0.001) were significantly higher in fetuses with CHD when compared to healthy controls (Table 3, Fig 1). The median MCA PI MoM (0.93 vs 0.97; p = 0.003) and CPR MoM (0.84 vs 0.95; p<0.001) were significantly lower in fetuses with CHD than controls (Table 3, Fig 1). The percentage of low CPR <0.6MoM cases (11.7% vs 2.5%; p<0.001) were significantly higher in the fetal CHD cohort.

**Table 3:** Biometry and Doppler parameters in the study groups.

	CHD (n = 153)	Controls (n = 658)	p value
**Mid-pregnancy uterine Doppler**			
Gestation at Doppler (weeks)	21.86 (21.14–22.93)	21.86(21.57–22.29)	0.947
Mean uterine artery PI multiple of median (MoM)	0.90 (0.73–1.06)	0.83 (0.68–0.98)	0.006
**Third trimester fetal biometry assessment**			
Gestation at ultrasound (weeks)	31.00 (22.29–34.29)	36.00 (28.29–37.14)	<0.001
Biparietal diameter centile	36.23 (17.18–62.12)	47.87 (27.49–67.89)	0.004
Head circumference (HC) centile	45.43 (22.01–66.27)	57.07 (37.43–71.76)	<0.001
Abdominal circumference (AC) centile	51.17 (25.89–69.74)	55.71 (39.37–71.03)	0.014
Estimated fetal weight (EFW) centile	33.60 (0.13–68.28)	56.73 (19.71–78.14)	<0.001
EFW <10^th^ centile	35.3 (%)	22.3 (%)	<0.001
**Fetal Doppler assessment**			
Umbilical artery PI MoM	1.05 (0.89–1.18)	0.97 (0.86–1.08)	<0.001
Middle cerebral artery PI MoM	0.93 (0.80–1.05)	0.97(0.87–1.09)	0.003
Umbilical cerebral ratio MoM	1.11 (0.93–1.36)	0.99 (0.84–1.15)	<0.001
Cerebroplacental ratio MoM	0.84 (0.69–1.01)	0.95 (0.81–1.12)	<0.001
Cerebroplacental ratio MoM <0.6	11.7 (%)	2.5 (%)	<0.001

The values are presented as number (%) or Median (interquartile range). Group comparisons were made with Mann-Whitney-U, Chi-Square or Fisher’s exact where appropriate.

Subgroup analysis of UtA PI MoM values according to the expected blood supply to the brain (low placental supply and mixed placental and systemic supply) revealed non- significant differences (low placental supply median UtA MoM 0.90, Interquartile range- IQR 0.73–1.07; mixed placental and systemic supply, median UtA PI MoM 0.91, IQR 0.75–1.06; p = 0.653).

## Discussion

### Summary of study findings

The findings of this study demonstrate that mid-gestational uterine artery PI indices were significantly higher, fetal biometry in the third trimester was significantly smaller and fetal Doppler indices were suggestive of uteroplacental dysfunction and fetal hypoxemia in fetuses with CHD compared to normal pregnancies.

### Clinical and research implications

Significantly higher second trimester mean uterine artery PI in fetuses affected by CHD, as demonstrated in this study, is related to impaired placentation. In combination with significantly lower birth weight centiles of CHD cases and lower MCA Doppler indices, higher second trimester mean uterine artery PI is indicative of redistribution suggestive of fetal hypoxemia; all these findings support uteroplacental dysfunction interrelating to growth restriction in CHD fetuses. Our findings are consistent with those by Ruiz et al., who demonstrated that the uterine artery PI is greater than the 95th centile in 61% of 115 CHD pregnancies. These results were again suggestive of uteroplacental dysfunction, even though the authors included a smaller number of CHD cases, did not include an appropriate control group and reported only on trends with gestational age [18].

Although the exact underlying mechanism leading to uteroplacental dysfunction is not yet fully understood, the association of the latter with fetal CHD is supported by the fact that the placenta and the fetal heart form concurrently at around day 21 of gestation[19]. Moreover placental and heart development share common pathways including the Notch and Wnt pathway which regulate both the specification of cardiomyocites and extra villous throphoblast invasion[20, 21]. Vascular cell adhesion protein (VCAM) 1, which mediates the adhesion of lymphocytes and monocytes to the vascular endothelium, shows lower levels in babies with fetal growth restriction[22]. A disruption of VCAM 1 described in a mouse model results in impaired placentation leading to both fetal demise and the presence of severe malformations of the fetal heart [23, 24].

As mentioned earlier, Llurba et al. [10] reported an imbalance in the sFlt-1 (soluble fms-like tyrosine kinase 1) and placental like growth factor (PlGF) levels in pregnant women carrying fetuses with CHD–a recognised finding in fetal growth restriction [25]. They suggested that uteroplacental dysfunction and resulting fetal hypoxemia might proceed to the subsequent formation of cardiac abnormalities in these fetuses as well as the complications of preeclampsia and fetal growth restriction [10]. This hypothesis is supported by animal studies on zebra fish embryos where a blockage of the VEGF receptor was associated with the formation of an abnormal fetal heart [26]. The imbalance of angiogenic factors seen in manifestations of uteroplacental dysfunction such as preeclampsia and fetal growth restriction, suggest an important primary or secondary role of uteroplacental function contributing to the development of CHD. Data on differences in uteroplacental perfusion in larger cohorts of CHD stratified by different subgroups, which might be at particular risk for placental insufficiency, are lacking. Analysis of our data in this study did not reveal significant differences between subgroups of CHD according to UtA PI MoM, suggesting no greater risk for the development of placental insufficiency in specific subgroups of CHD.

## Strengths and weaknesses

The strength of our study includes the relatively large sample size of fetuses with CHD and healthy fetuses with both fetal cardiac evaluation by a fetal cardiologist and uterine artery Doppler assessment. However, the main limitations are its retrospective nature and the non-consecutive case selection, the latter introducing the possibility of selection bias. Moreover, the number of fetuses with CHD was limited and subgroups of CHD were small. Therefore the analysis for differences in UtA PI MoM between different subgroups of CHD might have been underpowered and the lack of significance might be due to the small sample size. Larger prospective studies also analysing different subtypes of CHD are necessary to evaluate the role of uteroplacental perfusion and placentation as underlying mechanisms contributing to both CHD and preeclampsia in these patients. Furthermore the inclusion of maternal outcome parameters such as the prevalence of preeclampsia or chronic hypertension resulting in placental insufficiency would have been favourable. However, the study was not designed or adequately powered to proof a causal relationship between the presence of CHD and preeclampsia as described by Boyd et al [27] in a cohort of two million pregnant women, hence a regression analysis for preeclampsia was not performed.

## Conclusion

In this study, we were able to demonstrate significant differences in uteroplacental perfusion indices in pregnant women with fetuses affected by CHD compared to healthy pregnant controls, suggesting a potential contributing role of impaired uteroplacental perfusion in the pathogenesis of fetal CHD. The additional findings of smaller fetuses and Doppler indices of relative fetal hypoxemia suggest that CHD pregnancies are characterized by impaired uteroplacental perfusion, a known risk factor for preeclampsia. Further research is necessary to clarify the underlying mechanisms leading to fetal CHD, with a special focus on the role of impaired uteroplacental perfusion in women carrying a fetus with CHD.

## Supporting information

S1 File List of variables. (DOCX)
