# Supplementary material for: Evidence for uteroplacental malperfusion in fetuses with major congenital heart defects
Source: PLoS One. 2020 Feb 5;15(2):e0226741. doi: 10.1371/journal.pone.0226741 (PMC7001956; doi:10.1371/journal.pone.0226741)
Supplement: S1 File — (DOCX) [file pone.0226741.s001.docx]

List of variables

- Group: cases and controls
- Congenital heart disease Subgroup
- Age
- ethnicity
- Gestational age at fetal cardiac assessment
- Gestational age at uterine artery doppler assessment
- right uterine artery Pulsatility index
- left uterine artery Pulsatility index
- right uterine artery Multiple of the median
- left uterine artery Multiple of the median
- Mean uterine artery doppler Pulsatility index
- Mean multiple of the median uterine artery doppler
- Gestational age at third trimester doppler
- Umbilical artery Doppler Pulsatility index
- Umbilical artery Doppler Multiple of the Median
- Middle cerebral artery Doppler Pulsatility index
- Middle cerebral artery Doppler Multiple of the Median
- Cerebroplacental Ratio Pulsatility index
- Cerebroplacental Ratio Multiple of the Median
- Biparietal diameter
- Abdominal circumference
- Femur length
- Estimated fetal weight
- Estimated fetal weight centile
- outcome
- Gestational age at delivery
- Sex
- Birth weight
- Birthweight centile
- Mode of delivery
